# Sensory preferences and requirements amongst Swedish older adults with motoric eating difficulties

**DOI:** 10.29219/fnr.v66.8269

**Published:** 2022-10-25

**Authors:** Sarah Forsberg, Wender Bredie, Karin Wendin

**Affiliations:** 1 Department of Food and Meal Science and the Research Environment MEAL, Faculty of Natural Science, Kristianstad University, Kristianstad, Sweden; 2 Department of Food Science, Section for Food Design and Consumer Behaviour, University of Copenhagen, Frederiksberg C, Denmark

**Keywords:** motoric eating difficulties, older adults, product development, finger foods, sensory preferences and requirements, check-all-that-apply, summative content analysis

## Abstract

**Background:**

Finger foods, foods that can be eaten without cutlery, may be a strategy to increase autonomy and food intake amongst older adults with motoric eating difficulties. In order to develop optimal finger foods, knowledge about sensory preferences and requirements in the target population is needed.

**Objective:**

To assess sensory preferences and requirements amongst Swedish older adults with motoric eating difficulties.

**Design:**

Check-all-that-apply (CATA), a methodology that does not require much cognitive effort, was used to collect survey data about sensory preferences and requirements for everyday meals from 15 older adults with motoric eating difficulties. The CATA-questionnaire was structured according to the Swedish meal order (breakfast, lunch, dinner, snack and fika) and consisted of 29 attributes compiled through a literature review.

**Results:**

Through both qualitative and quantitative data analysis, it was found that flavourful, flavour intensity, spicy and both Swedish and ethnic flavours were important attributes related to food flavour. Although most participants preferred crispy and coarse textures, a few participants found soft, smooth and fine textures important. Moreover, colourful meals and serving components separated on the plate were important for the appearance of lunch and dinner.

**Discussion:**

A diverse range of flavours, flavour enhancement and a balance between the basic tastes and spiciness may increase the flavour perception. Finger foods should be offered in the full range of flavours available to the general older adults’ population. The variation in the demand for texture may be related to chewing and swallowing difficulties, and textures that require moderate chewing and easy swallowing are, therefore, optimal for finger foods. Separating meal components on the plate may make it easier to distinguish the components.

**Conclusion:**

Chemosensory impairments, chewing and swallowing difficulties, and visual disturbances are important to consider in the development of finger foods.

## Popular scientific summary

- A diverse range of flavours, flavour enhancement and a balance between the basic tastes and spiciness may increase the flavour perception.
- The development of finger foods should consider the full range of flavours available to the general older adults’ population.
- Fine, soft and smooth textures requiring moderate chewing and easy swallowing are optimal for finger foods.
- Serving meal components separately on the plate is important for the appearance of lunch and dinner.

Motoric difficulties, such as tremors, rigidity, functional impairments, pain and weakness in the hands and fingers, may influence the autonomy and food intake of older adults negatively since their ability to prepare food, manage cutlery and transport food to the mouth may be reduced (1–3). In a recent study, motoric eating difficulties were found to be the most severe forms of eating difficulties (4). Additionally, older adults with motoric eating difficulties were more likely to have other eating difficulties, such as with chewing and swallowing, and reduced appetite and energy (4). Westergren et al. (5) found that motoric eating difficulties were significantly associated with assisted eating, and that 46% of the participants were also either at risk of being malnourished or had suspected or manifest malnutrition (5).

The use of eating aids, such as modified cutlery, sip cups and plates with high edges, is one way to help facilitate independent eating amongst persons with motoric eating difficulties. Nyberg et al. (6) found that eating aids were valuable for maintaining proper eating behaviour; however, eating aids were not commonly used by the participants. Instead, the participants adopted their own strategies, such as using both hands or a straw when drinking and cutting the food into pieces and eating it with a spoon (6). For older adults with minor or moderate eating difficulties, eating aids, forks and spoons may be helpful when adjusting to new circumstances. However, for those with major eating difficulties, finger foods that are easy to grip and transport from the plate to the mouth may be more ideal (7).

Finger foods have been defined as foods that do not require cutlery or could be eaten easily with the hands (8). Finger foods of high acceptability may also improve dignity in meal situations and, in turn, quality of life for older adults. Older adults’ perceptions and attitudes have been investigated in a previous study and resulted in insights about acceptability of such foods in the target population. However, for finger foods to have the desired effect, nutritional and sensory aspects also have to be taken into consideration. Knowledge about individual preferences (9) and sensory perception (10) has been found to be important when tailoring meals of high acceptability for older adults. Research has shown that a decline in sensory perception of olfaction and gustation is common amongst persons with Parkinson’s disease and after a stroke (11–14), but more knowledge is needed about how taste, smell, temperature, colour and texture influence the palatability of foods and food enjoyment amongst older adults with motoric eating difficulties. However, involving the target population in research can be challenging due to cognitive decline and physiological limitations.

The purpose of this study is to assess sensory preferences and requirements amongst Swedish older adults with motoric eating difficulties.

## Material and methods

### Research design and methodology

**Fig. 1:**
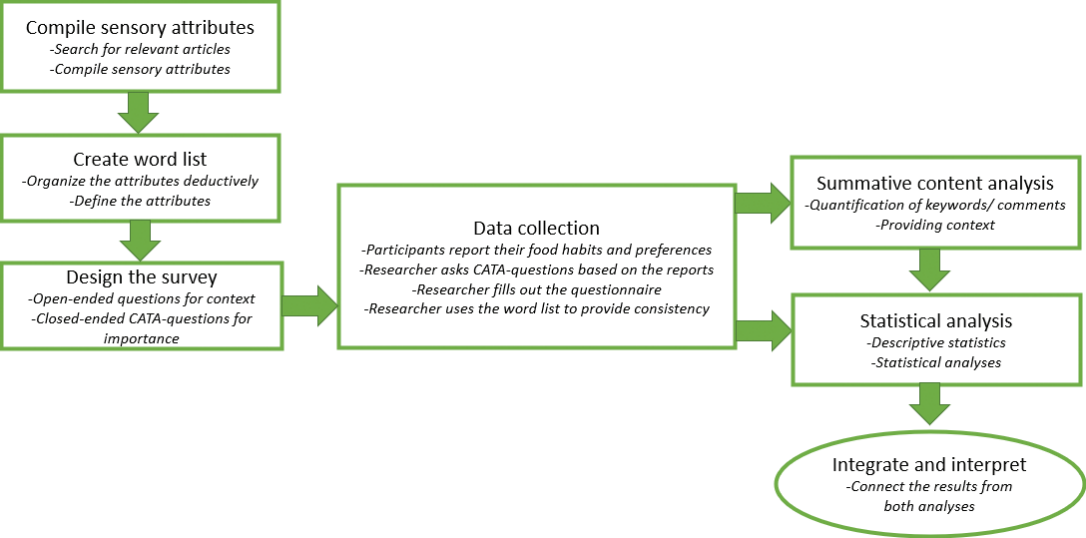
Overview of the planning, data collection and data analysis of the study.

A mixed method, convergent design was chosen for this study since both qualitative and quantitative data were needed to answer the research question. A convergent design merges data to gain information about the research problem from multiple angles (15). In this study, to facilitate the analysis of the survey data, the qualitative data were needed to provide context for the quantitative data (Fig. 1). Without a description of the food and meal preferences amongst the participants, it would be difficult to understand and relate the sensory attributes found in the survey.

### Check-all-that-apply

Check-all-that-apply (CATA) is a consumer-friendly methodology used to obtain rapid product profiles from consumers (16). A CATA-questionnaire with a list of terms is presented where the consumers are able to indicate multiple words or phrases that apply to and describe their experiences of the product or sample being evaluated (17). This can include sensory attributes, hedonic and emotional responses or purchase intentions that the consumers associate with the product or sample (16). However, CATA has been seldom used with older adults (18).

### Compile sensory attributes

Relevant articles and scientific reports were reviewed to compile sensory attributes for the CATA questionnaire. Articles were searched in the database Summon, obtained through reference lists of other studies in the field, and some articles and reports were already known from the field. Twenty articles and scientific reports concerning the preferences for and acceptability of food and meals amongst older adults in a Scandinavian context were chosen and assessed; eight articles (8, 19–25) were included in the review (Table 1). The sensory attributes found in the articles were documented.

**Table 1:** Overview of the literature review and collected sensory attributes

Author and year	Sensory attributes
Forslin (19)	Appetising appearance Optimal texture Chewiness Hard Soft Warm food Aromatic odours Tasty Crispy Distinct flavours
Hall and Wendin (20)	Particle size Fatty Juicy Creamy Smooth Intense taste and flavour Firm Soft Coarse texture Appetising appearance Tasty
Giacalone et al. (21)	Overall taste and flavour Cold foods Saltiness Sweetness Odour intensity Variation of flavours Tasty
Höglund et al. (22)	Colourful appearance Served separately on the plate Well-seasoned Flavour intensity Optimal sauce consistency Visible components Tasty
Armanyarahmadi and Wendin (23)	Spicy food Flavour Texture Well-seasoned New flavours Warm food
Okkels et al. (24)	Texture: minced and moist and puréed Temperature: warm, cold and frozen Appearance: in layers-sprinkled Basic tastes: sweet, sour and salty Colourful: different colours Several flavours
Edfors and Westergren (9)	Appealing appearance Not spicy Well-known traditional flavours (Swedish) Modern and unfamiliar flavours (ethnic flavours) Texture: properly cooked Flavourful Chewy
Nordlander et al. (25)	Culturally adapted food (Swedish) Tasty/tasteful Savoury foods Colourful Well-seasoned Carefully salted Too spicy Too sour Unbalanced Tasteless Sprinkled with herbs Too hard Unappetising appearance Overcooked Familiar

### Create word list

Based on the literature review, the sensory attributes were structured in a word list according to appearance, flavour, texture, temperature and odour (Table 2). Sensory attributes of similar nature were grouped together and given a label. In total, 29 attributes were compiled and defined using a comprehensive contemporary Swedish-language encyclopaedia to make the survey as standardised as possible.

**Table 2:** Overview of the sensory attributes used in the CATA-questionnaire

Appearance	Flavour	Texture	Temperature	Odour
Light colour	Flavour intensity	Fatty	Cold	Intense smell
Dark colour	Flavourful	Juicy	Warm	Aromatic
Colourful	Umami	Creamy		
In layers	Sweet	Crispy		
Mixed on the plate	Salty	Hard		
Separated on the plate	Sour	Soft		
	Swedish flavours	Firm		
	Ethnic flavours	Smooth		
	Spicy	Fine texture		
		Coarse texture		

### Design the survey

A survey was created in the software EyeQuestion® (26). The first part of the questionnaire consisted of demographic questions about gender, age, marital status, diagnosis and sensory function. The second part of the questionnaire was structured according to traditional Swedish daily meals, with a section each for breakfast, lunch, dinner, snacks and fika (coffee and cake). Each section started with an open-ended question, so that the participants could report their food preferences for each meal, followed by a list of the 29 sensory attributes (CATA terms).

### Recruitment

The recruitment was conducted with support from representatives from the Scanian Parkinson coalition and the Network for Eating and Nutrition (NEN) (27). NEN is a platform for cooperation over organisational borders in healthcare sectors in the northeast of the Swedish province of Scania (27). An information letter was sent out to the representatives of the Scanian Parkinson coalition and NEN, who then forwarded it on within their organisations and recruited suitable participants. Inclusion criteria required that the older adults were 65 years or older, had some type of motoric eating difficulty (tremor, rigidity, coordination problems, paresis, etcetera that influenced their ability to eat with cutlery) and were able to communicate in Swedish and consent or assent to an interview. An informed and written consent was obtained before any contact was established with the participants and any appointments were booked. Nineteen participants gave their consent to participate in the study; however, three dropped out and one was unable to participate due to health concerns. Thus, 15 older adults participated in this study.

### Data collection

Data were collected during individual short interviews with the participants in their own homes, including own home and nursing home. Twelve of the participants had previously participated in individual interviews for another study about perceptions and attitudes about eating with their fingers (7). The interviews were conducted as part of a conversation over a cup of coffee, and the survey was digitally administrated. The first author asked the questions, documented the participants’ meal preferences (open-ended questions) and checked off the sensory attributes that the participants considered important for each meal (CATA questionnaire). A standardised word list was used to describe the attributes to ensure that the participants understood the meaning of the attributes in order to respond accordingly. The interviews lasted approximately 10–20 min, and six participants received support from their spouse during the interview. The interviews were conducted from May 2019 to February 2020.

### Participants

This study was carried out with 15 older adults aged 65–85 years with eating difficulties (six female and nine male). Nine of the participants were diagnosed with Parkinson’s disease, two had atypical Parkinsonism and four had suffered strokes. In addition, 10 participants reported that they suffered from decreased sensory functions (Table 3). Four participants lived in a nursing home and 11 participants in their own home.

**Table 3:** Overview of the demographics of the participants, frequency (F) and percentages (%)

*N* = 15	*F*	%
**Gender**		
Female	6	40
Male	9	60
**Age**		
81–85 years	3	20
76–80 years	5	33
71–75 years	4	27
65–70 years	3	20
**Marital status**		
Single	3	20
Married/domestic partnership	7	46
Widowed	4	26
Live-apart partnership	1	7
**Accommodation**		
Nursing home	4	73
Ordinary home	11	27
**Diagnosis**		
Parkinson’s disease	9	73
Stroke	4	26
Atypical Parkinsonism	2	1
**Motoric eating difficlties**		
Minor	5	33
Moderate	6	40
Major	4	27
**Sensory function**		
Yes, decreased sense of taste	3	20
Yes, decreased sense of smell	4	27
Yes, both decreased sense of taste and smell	3	20
No, neither	5	33

The eating difficulties were categorised into minor, moderate and major based on the participant’s ability to handle cutlery. Participants with the ability to handle a knife and fork were categorised as having minor eating difficulties, participants with the ability to handle a fork and a spoon as having moderate eating difficulties and participants who ate with a spoon or a fork complemented by their fingers as having major eating difficulties (7).

### Data analysis

Descriptive statistics were analysed to describe the sample and the importance of the sensory attributes. Cochran’s Q test was conducted to assess the difference in proportion between related samples. A correspondence analysis was then conducted using CATA counts weighted variables to check for relationships. The data were analysed using IBM SPSS Statistics (Version 26). The level of significance was set to *P*-value ≤ 0.05 for all statistical analyses.

The answers to the open-ended questions were analysed with inspiration of summative content analysis (28). The transcript of the survey output was printed out, and the answers to the open-ended questions for each meal were assessed individually (breakfast, lunch, dinner, snack and fika). Thereafter, the different meal components and the preferred food items/meal components were highlighted in different colours and quantified based on the number of times they were mentioned. The qualitative and quantitative data were thereafter converged, by connecting the sensory attributes to the reported meal components for each meal.

### Ethical considerations

This study was performed in accordance with the Helsinki Declaration of Ethical Principles, including informed and written consent (29). Data were handled according to the guidelines of GDPR (General Data Protection Regulation) (30).

## Results

### Self-reported meal preferences

#### Breakfast

The participants reported that they ate a substantial breakfast consisting of several types of components (Table 4). Fourteen participants reported that they ate open sandwiches for breakfast, preferably made with dark and high fibre bread, with toppings such as cheese and ham with tomatoes and cucumber (Table 4). Ten participants ate yoghurt with cereal and/or berries, five ate porridge and four ate eggs (Table 4).

**Table 4:** Overview of the reported food preferences

Breakfast	#	Lunch	#	Dinner	#	Snack	#	Fika	#
Open sandwich Yoghurt Porridge Egg Ham Cheese Liver paste Dark bread Light bread Tomatoes Cucumber Bell peppers Banana	14 10 5 4 3 9 1 9 3 6 3 1 1	Cooked hot meal* Light meal** Late breakfast Swedish cuisine Soup Crepes Fish Rice pudding Porridge Yoghurt Open sandwich Banana Meat, potatoes and gravy Leftovers Salad Vegetables	10 3 2 2 1 1 1 1 2 2 2 2 7 1 2 2	Cooked hot meal* Light meal** Swedish cuisine Meat, potatoes and gravy Porridge Pickled herring Egg Omelettes Open sandwich Sandwich cake Crisp bread Salad Rosehip soup Fruit fools Raisins, seeds and nuts Vegetables Yoghurt	7 8 2 5 3 1 3 2 5 1 1 4 2 1 2 1 1	Fruits Open sandwich Shrimps Hamburger Coffee/cookies Nutritional supplements	1 0 3 2 1 2 1	Coffee Cookies Cake Fruits Open sandwich Chocolate Ice-cream Nut	7 11 2 2 2 4 1 3

* Cooked hot meal: consisted of meat, potatoes and gravy.

** Light lunch/dinners consisted of eggs, omelettes, soups, salads, sandwiches and porridge.

# How many times the component was mentioned in the open questions.

#### Lunch and dinner

Ten participants reported that they ate cooked, hot meals for lunch (meat, potatoes and gravy), whilst five ate lighter meals or late breakfast consisting of, e.g. open sandwiches, eggs, yoghurt, rice pudding, banana or porridge (Table 4). Seven participants ate cooked, hot meals (meat, potatoes and gravy) for dinner, whilst eight ate lighter meals consisting of, e.g. open sandwiches, omelettes, salad, crisp bread, eggs, rosehip soup, yoghurt or porridge (Table 4).

Six participants appreciated a balance between the basic flavours and six participants appreciated condiments such as lingonberries or pickled cucumbers and beetroot with their meals (Table 5). Seven participants wanted a lot of gravy as it made the food moist and easier to swallow. Ten participants reported that they appreciated a variation in texture of the components, and two participants reported that they needed their meat cut into pieces (Table 5).

**Table 5:** Overview of comments about sensory attributes that were reported in the open-ended comment section

Appearance	#	Flavor	#	Texture	#	Temperature	#
Trimmings – color No puréed foods	2 1	Pickled trimmings – acidity Vinaigrette Balance – basic tastes Smoked foods	6 2 6 3	Lots of gravy – moisture Variation in texture Meat in pieces Not hard meat	7 10 2 1	Cold – easy to swallow	1

# How many times the component was mentioned in the open questions.

#### Snack and fika

Snacks consisted of fresh fruit and open sandwiches, whilst fika was considered to be a cup of coffee with cookies or cake (Table 4). Cakes and cookies with chocolate and nuts were especially appreciated (Table 5).

### Important sensory attributes

**Fig. 2:**
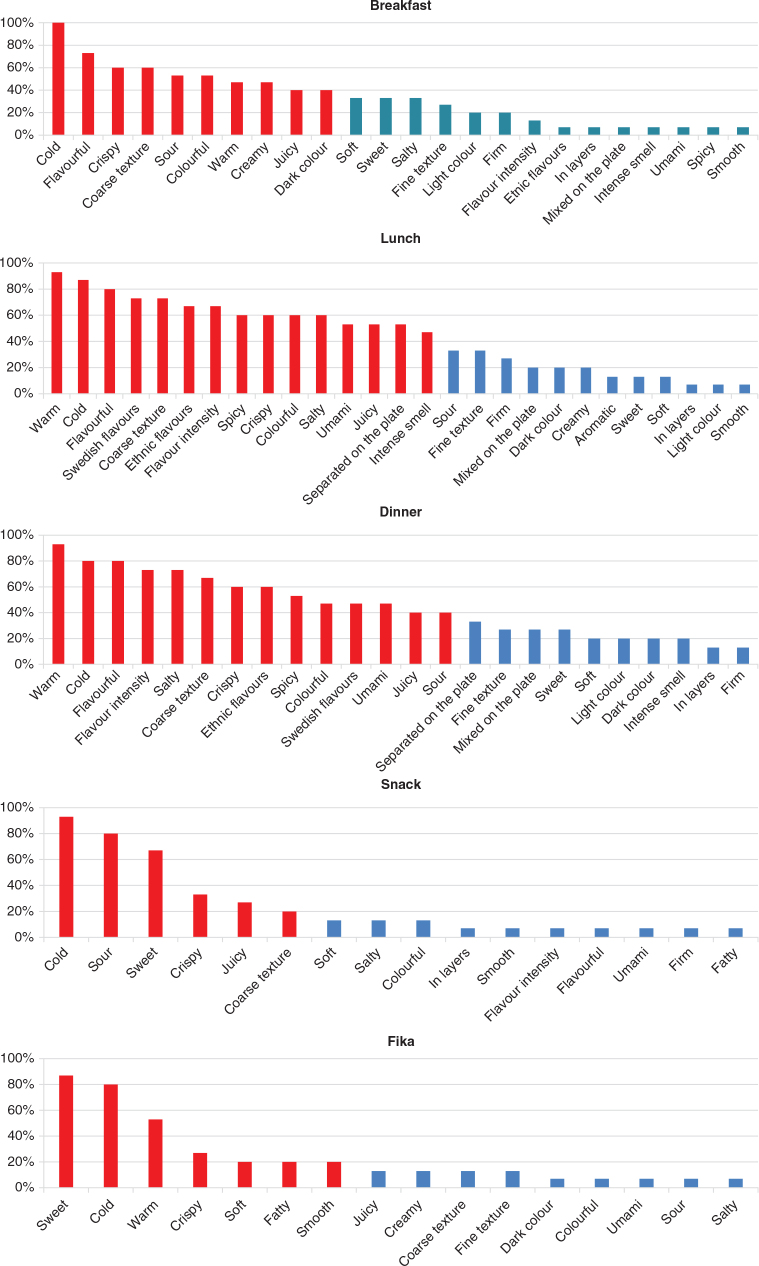
An overview of the important sensory attributes for all the meals. Most important attributes for breakfast, lunch and dinner, range 100–40% in subsequent order (marked in red); less important attributes, range 39–1% (marked in blue). Most important attributes for snacks and fika, range 100–20% in subsequent order (marked in red); less important attributes, range 19–1% (marked in blue).

The most important sensory attributes for breakfast were cold, flavourful, crispy, coarse texture, sour, colourful, warm, creamy, juicy and dark colour (Fig. 2). The most important sensory attributes for lunch were warm, cold, flavourful, Swedish flavours, coarse texture, ethnic flavours, flavour intensity, spicy, crispy, colourful, salty, umami, juicy, separated on the plate and intense smell. The most important sensory attributes for dinner were warm, cold, flavourful, flavour intensity, salty, coarse texture, crispy, ethnic flavours, spicy, colourful, umami, Swedish flavours, sour and juicy (Fig. 2). The most important sensory attributes for snacks were cold, sour and sweet, whilst the most important sensory attributes for fika were sweet, cold and warm (Fig. 2).

### Differences between meals

Significant differences between the meals for each attribute included in the CATA were identified. The distribution of the responses for light colour, in layers, aromatic, juicy, crispy, soft, firm, smooth and cold, was the same for all the meals. However, the remaining attributes differed significantly between the meals (Table 6).

**Table 6:** Overview of the frequencies [*F*] of the attributes in breakfast (*B*), lunch (*L*), dinner (*D*), snacks (*S*) and fika (*F*)

Sensory properties	*[F]B*	*[F]L*	*[F]D*	*[F]S*	*[F]F*
Light colour	3	1	3	0	0
Dark colour	6^a^	3^abc^	3^abc^	0^bc^	1^c^
Colourful	8^a^	9^a^	7^a^	2^b^	1^b^
In layers	1	1	2	1	0
Mixed on the plate	1^ad^	3^bd^	4^b^	0^ac^	0^ac^
Separated on the plate	0^a^	8^bc^	5d^e^	0^a^	0^a^
Intense smell	1^a^	7^b^	3^a^	0^a^	0^a^
Aromatic	0	2	0	0	0
Flavour intensity	2^a^	10^b^	11^b^	1^a^	0^a^
Flavourful	11^a^	12^a^	12^a^	1^b^	0^b^
Umami	1^a^	8^b^	7^b^	1^a^	1^a^
Sweet	5^ad^	2^ac^	4^ac^	10^d^	13^b^
Salty	5^ac^	9^c^	11^b^	2^a^	1^a^
Sour	8^ab^	5^b^	6^b^	12^ac^	1^d^
Swedish flavours	0^a^	11^b^	7^b^	0^a^	0^a^
Ethnic flavours	1^a^	10^b^	9^b^	0^a^	0^a^
Spicy	1^a^	9^b^	8^a^	0^a^	0^a^
Fatty	0^a^	0^a^	0^a^	1^a^	3^b^
Juicy	6	8	6	4	2
Creamy	7^a^	3^b^	0^b^	0^b^	2^b^
Crispy	9	9	9	5	4
Hard	0	0	0	0	0
Soft	5	2	3	2	3
Firm	3	4	2	1	0
Smooth	1	1	0	1	3
Fine texture	4^a^	5^a^	4^a^	0^b^	2^a^
Coarse texture	9^a^	11^a^	10^a^	3^b^	2^b^
Cold	15	13	12	14	12
Warm	7^ab^	14^c^	14^c^	2^d^	8^a^

Different letters for each given sensory property indicate significant differences between meals at *P* < 0.05.

**Fig. 3:**
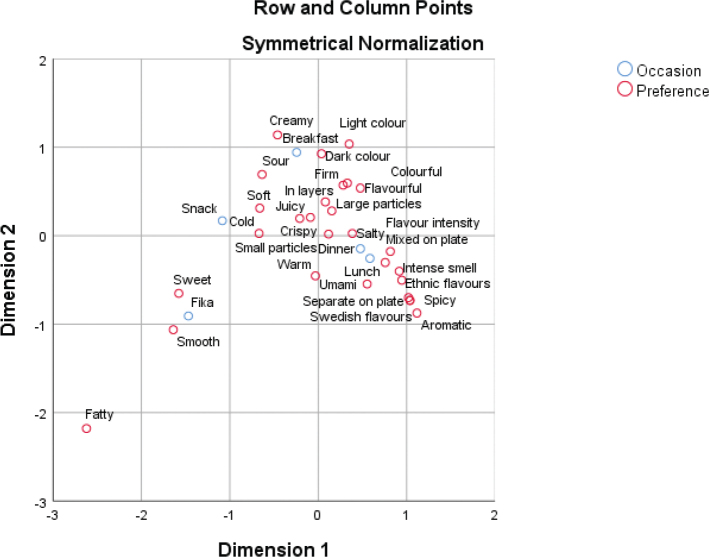
Explained variance for dimension 1 (62.3%) and dimension 2 (19.5%) of the correspondence analysis using the observed proportions from the CATA responses.

The correspondence analysis estimated the relationship between the meals and the attributes. The plot shows the first two dimensions (Fig. 3), which capture 81.8% of the variance, and the third dimension which adds 13.8%, in total 96.6% of the variance. Since the remaining dimensions only account for 4.4%, they are not considered relevant. The correspondence analysis confirms the results from the Cochran’s Q test.

## Discussion

### Sensory preferences and requirements

This study builds on qualitative data complemented with results from quantitative data. Although the sample population was small, the qualitative findings supported the quantitative and, thus, the internal validity of the CATA methodology.

#### Food flavour

Flavourful meals were important for acceptability of breakfast, lunch and dinner by the participants (Fig. 2 and Table 6). Flavourful in this study was defined as a diverse range of flavours as part of a meal. Hollis and Henry (31) found that older adults consumed significantly more food when they were presented with varied meals rather than a series of identical foods. Meals combined with diverse flavours and meal components may, therefore, stimulate appetite and increase food intake due to sensory-specific satiety being avoided, and promote a more balanced diet.

Flavour intensity was found to be significantly more important for lunch and dinner than for breakfast, snacks and fika (Fig. 2 and Table 6). This may be due to the flavour complexity in more substantial and cooked meals. Several participants reported that a balance between the basic tastes was preferred in cooked meals. According to Klosse et al. (32), flavours are well balanced in palatable foods. Balancing flavours to create a harmonious taste may, therefore, enhance the overall flavour intensity. In Sweden, savoury dishes are traditionally balanced with pickled sour-sweet condiments and lingonberries, although the specific condiment combinations will differ in other cultural contexts.

A majority of the participants experienced chemosensory losses and may, therefore, perceive flavours less intense. This may explain why flavour intensity and spicy were considered important. A decline in sensory functions including the chemical senses is frequently occurring in older age (33–35) and may include losses in sensitivity to taste, olfactory and trigeminal stimuli (36, 37) and low recognition of salty, bitter, sour and umami tastes amongst older adults compared with young adults (38). Yet, normal and gradual sensory losses do not cause a reduced food liking in older adults, as they continuously adjust to their diminished perception (39). Moreover, Honnes de Lichtenberg Broge et al. (40) showed that despite the decline in intensity perception for everyday food odours, the liking for the food odours, especially the savoury food odours, largely remained intact. However, nine participants in this study were diagnosed with Parkinson’s disease, a degenerative disease with severe sensory disturbances (41) where olfactory impairment is a part of the clinical diagnosis (42). Flavour enhancement and aromas may, therefore, improve the palatability of meals for this group. Moreover, spicy food may have a positive impact on flavour perception, evoking activation of the trigeminus nerve and giving feelings of warmth, coolness and irritation (43). However, control over the spiciness needs attention as high levels of activation lead to pain sensation (44).

Both Swedish flavours associated with cooked warm meals and traditional ingredients and spices, and ethnic flavours associated with ingredients, spices and foods from cuisines around the world were appreciated for lunch and dinner (Fig. 2 and Table 6). Previous studies have found that Swedish older adults prefer home-cooked and traditional dishes, and familiar spices that they had in their childhood (9, 25, 45). Also, Hall (46) found that eating habits amongst Swedish older adults had not changed much in older age. However, some studies indicate that dietary patterns amongst Swedish older adults have changed over the years (47). For example, Swedish 70-year-olds food patterns correspond to Mediterranean dietary patterns (47), and convenience foods, such as pizza, hamburgers, kebabs, tacos and hot dogs from international cuisines, were enjoyed and frequently eaten by adults with motoric eating difficulties aged 65 years and older (7). Since older adults with motoric difficulties appear to have similar meal preferences, the development of finger foods should consider the full range of flavours available to the general older adult population.

The distribution of responses for snack and fika was similar (Fig. 2), and the participants considered these meals to be more or less the same. However, some differences appeared. Cold, sour and sweet were the most important sensory attributes for snacks, whilst sweet, cold and warm were most important for fika (Fig. 2). Snacks consisted mainly of fruits and sandwiches, whilst fika consisted of coffee and something sweet.

#### Texture

A majority of the participants appreciated variations in texture. According to Klosse et al. (31), contrast in mouthfeel, a combination of crispy and crunchy together with juicy, creamy and moist, is crucial for the palatability of foods. Although a hard texture was not appreciated, most participants preferred crispy and coarse textures over smooth and fine textures (Fig. 2 and Table 6). This indicates that the majority of the participants did not have chewing and swallowing difficulties, and that regular foods with a variety of textures are optimal for this group. However, a few participants found soft, smooth and fine textures important, which indicates a variability in texture perception amongst older adults with motoric eating difficulties that is important to acknowledge (Fig. 2 and Table 6).

According to Westergren and Melgaard (4), older adults with motoric eating difficulties are more likely to have other eating difficulties, such as with chewing and swallowing. Chewing and swallowing difficulties are common conditions after a stroke (1, 2, 48) and in the later stages of Parkinson’s disease (49). For persons with atypical Parkinsonism, deglutition can be severely impaired even during early stages of the disease (49, 50). Two of the participants in the study suffered from atypical Parkinsonism, which may explain the variation in texture perception. According to the study by Forsberg et al. (7), older adults with minor and moderate motoric eating difficulties were still able to eat with cutlery or spoon and/or fork, whilst eating with the fingers was more common and acceptable amongst older adults with major motoric eating difficulties. Since chewing and swallowing difficulties are common in this population group, fine, soft and smooth textures that require moderate chewing and are safe to swallow are optimal for finger foods.

#### Appearance

Colourful was found to be a significantly more important attribute for breakfast, lunch and dinner than for snacks and fika (Fig. 2 and Table 6). The participants appreciated colourful presentations such as sandwiches decorated with vegetables, and yoghurt and porridge served with berries. This corresponds to several studies, for example Mahadevan et al. (51), which found that a variety of colours and garnishes were important for acceptability by older adults. Also, visual arrangements of minor food components like toppings have been shown to stimulate appetite in elderly (52). Moreover, colour has a profound effect on taste perception and plays a critical role in food acceptance (53); serving vegetables in a variety of colours may have a positive effect on food intake.

Serving meals where the components are placed separately on the plate was also found to be significantly most important for the appearance of lunches and also highly important for dinners (Fig. 2 and Table 6). This corresponds to previous studies by Höglund et al. (22) and Hall and Wendin (20). Separating the components on the plate makes it easier to distinguish the meal components (20). This may be particularly important for those with Parkinson’s disease since the disease is associated with visual symptoms such as poor acuity, especially at low contrast and vision blurred for colour stimuli (54).

### Strengths and limitations

#### Recruitment

Although the recruitment process was supported by the Scanian Parkinson coalition and NEN, only 15 older adults completed the survey. However, the sample population for this study is hard to reach due to disease and functional impairments. Six participants suffered from decreased cognitive ability but were able to adequately participate in this study with the support of their spouses. Including these participants was a strength since the voices of the target group cannot be complemented with other older adult populations.

According to Berkman et al. (55), including family members and other caregivers as proxies may help to obtain the perceptions and experiences of older adults, although the use of proxy respondents may also affect the validity of the study (56). However, since the spouses cared for and assisted them in their everyday lives and during meals, they had knowledge about their sensory preferences and requirements. The answers given by the participants who were supported by their spouses should, therefore, be considered reliable.

Recruiting other older adult populations in order to increase the sample size was not an option since the result would not be representative of older adults with motoric eating difficulties. However, there is a risk that participants suffering from minor and moderate eating difficulties may not be representative for the target group in need of finger foods. That would explain the variability in demand for texture found in this study. Further studies with older adults with major motoric eating difficulties are, therefore, needed.

#### CATA

This study showed that CATA is an easy method to apply in research with older adults as it does not require as much cognitive effort. In this study, the attributes were not focused on specific products but rather on sensory preferences and requirements of foods eaten at every meal during the day (breakfast, lunch, dinner, snack and fika). This approach has not been used previously for CATA. One limitation is that the foods eaten at every meal differ between people, and it can be difficult to apply the sensory attributes to specific food items. To facilitate this, it was important to obtain information about food preferences and eating patterns for all meals, to be able to put the sensory attributes into a context. Summative content analysis was used to create context for the analysis, and by counting meal components, a clear picture of the foods eaten at every meal was obtained. The food preferences and food habits reported in this study corresponded with previous research about Swedish eating patterns (46, 57, 58). To assure loss of information, the participants were also able to add or expand their views of the attributes in the open comments sections. This is a strength.

Another limitation is the binary response format as it does not allow measurement of the intensity of the attributes (59). Applying intensity measurements, such as Rate-All-That-Apply (60), may have offered more insights into differences between lunch and dinner, which were generally similar. However, the use of intensity scales was considered too advanced for the sample population since cognitive decline is common amongst older adults with diseases such as Parkinson’s disease.

There are several studies reporting the food preferences and food choices of older adults, but with no details of the specific sensory preferences and requirements of older adults with motoric eating difficulties. Knowledge about sensory preferences and requirements is vital to be able to develop attractive finger foods that older adults are willing to eat. Despite the small sample size, this study offers an opportunity to apply statistics that can guide the development of finger foods that cannot be achieved by qualitative data collection alone.

## Conclusions

This study found that a diverse range of flavours, flavour enhancement and a balance between the basic tastes and spiciness may increase the flavour perception, stimulate appetite and promote a more balanced diet amongst older adults with motoric eating difficulties. The development of finger foods should also consider the full range of flavours available to the general older adults’ population. This study also found a variability in texture perception that may be related to chewing and swallowing difficulties occurring in advanced stages of Parkinson’s disease and atypical Parkinsonism. Since finger foods may be more acceptable and beneficial for this population, fine, soft and smooth textures that require moderate chewing and easy swallowing are optimal. Finally, serving meal components separately on the plate may be important for lunch and dinner as it makes it easier to distinguish the components for those with visual symptoms. However, more research focusing on older adults with major eating difficulties are needed.
